# The molecular mechanisms and potential therapeutic implications of the crosstalk between DNA methylation and metabolic reprogramming in thyroid cancer

**DOI:** 10.1038/s41420-026-02981-8

**Published:** 2026-02-25

**Authors:** Tianying Zhang, Hengtong Han, Yating Zhang, Tingting Zhang, Libin Ma, Ze Yang, Yong-xun Zhao

**Affiliations:** 1 The First School of Clinical Medicine, Lanzhou University, Lanzhou, China; 2 The Seventh Department of General Surgery, Department of Thyroid Surgery, The First Hospital of Lanzhou University, Lanzhou, China

**Keywords:** Thyroid cancer, Cancer epigenetics, DNA methylation, Cancer metabolism, Oncogenesis

## Abstract

One of the fastest-growing malignant tumors in the world is thyroid cancer (TC), and there are currently no effective treatments for its aggressive subtypes, such as anaplastic carcinoma and radioactive iodine-refractory differentiated thyroid carcinoma. Recent investigations have shown that DNA methylation and metabolic reprogramming are not independent events, but rather create a closely interconnected, mutually reinforcing network of carcinogenic processes. On the one hand, metabolic reprogramming influences the methylation status of tumor suppressor genes and thyroid function genes by dynamically regulating the activity of DNA methyltransferases and demethylases through important metabolites (such as S-adenosylmethionine, or SAM, and α-KG) and oncogenic signaling pathways (like PI3K/AKT). Conversely, DNA methylation systematically remodels cellular glucose, lipid, and amino acid metabolism by directly silencing metabolic enzyme genes (such as FASN and GLS) and thyroid differentiation markers (such as NIS) to fulfill its proliferative demands. Tumor growth, treatment resistance, and the development of an immunosuppressive microenvironment are all fueled by this ongoing bidirectional interaction, which creates a self-reinforcing oncogenic cycle. As a result, the limitations of earlier discrete debates on DNA methylation or metabolic reprogramming are overcome in this review. To methodically clarify their crosstalk mechanisms, a theoretical framework based on the “DNA methylation-metabolism axis” is suggested. Additionally, it suggests multimodal therapy approaches that focus on this axis. Incorporating biomimetic delivery technologies, combined with epigenetic, metabolic, and immunotherapies, to lay the groundwork for comprehending TC causes and creating targeted treatments.

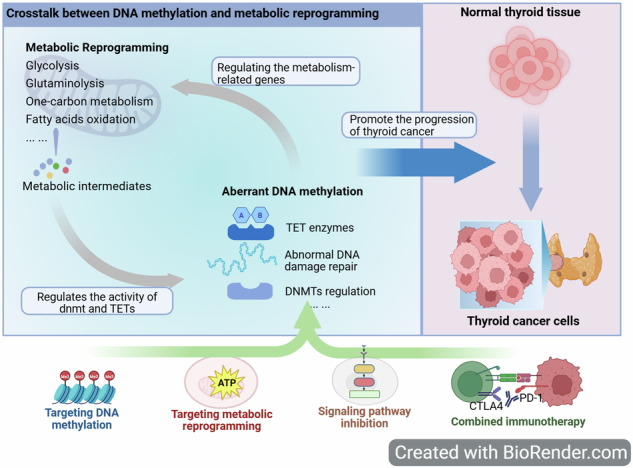

## Facts

- DNA methylation and metabolic reprogramming form a bidirectional, self-reinforcing loop that drives thyroid cancer progression and therapy resistance.
- Epigenetic silencing of genes like NIS is a key cause of radioiodine resistance, but durable reversal strategies are lacking.
- Combining epigenetic and metabolic drugs shows preclinical synergy, yet clinical translation faces toxicity, resistance, and biomarker challenges.
- Multi-omics and ctDNA analysis are crucial for addressing tumor heterogeneity and enabling precision therapy.
- Targeting the integrated “methylation-metabolism-immunity axis” is promising but requires better delivery systems and toxicity management.

## Questions

- How do metabolites precisely regulate DNMT/TET enzyme activity and specificity in thyroid cancer?
- Which key node in the crosstalk network is the primary driver of malignant traits like RAI resistance?
- How can we overcome toxicity and resistance to clinically translate epigenetic-metabolic combination therapies?

## Introduction

**Fig. 1:**
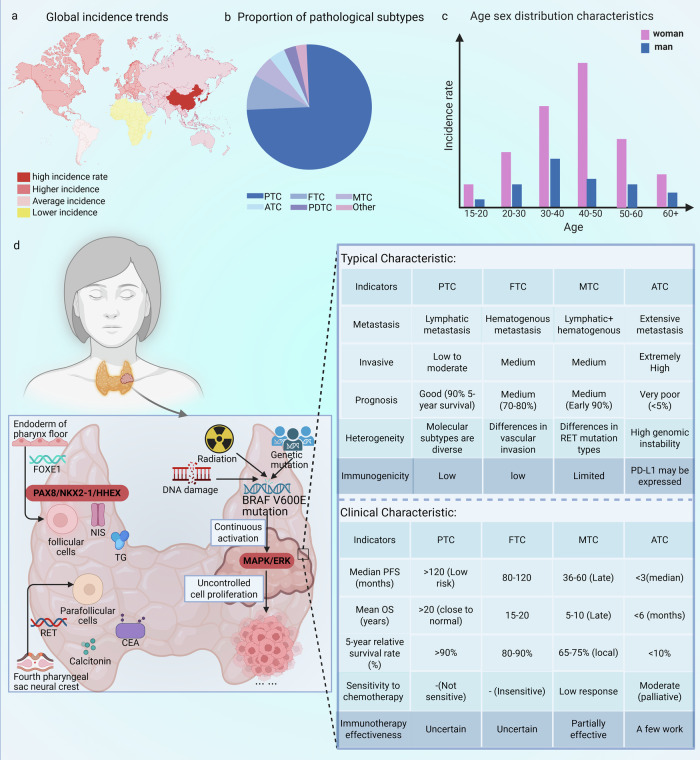
Conceptual diagram of the epidemiology, cellular origin, and clinical characteristics of thyroid cancer (TC). Thyroid cancer is unevenly distributed globally, with higher incidences in North America, Europe, and East Asia. The main risk factors include genetic susceptibility, radiation exposure, and impaired DNA repair mechanisms. PTC accounts for 80–85% of all TC subtypes. The proportion of female patients is higher than that of men (75–80%). The peak incidence occurs between the ages of 30 and 50. ATC is more common in elderly patients. Mutations in driver genes such as BRAF and RET play a key role in pathogenesis. Please see the attached table for details of clinical symptoms. Data Source: [1–3, 155]. Created with BioRender.com.

The most prevalent malignant tumor of the endocrine system is thyroid cancer (TC). According to the data from the US SEER database [1], TC is currently the ninth most common malignant tumor worldwide, and its incidence is continuously increasing. In terms of pathology, papillary thyroid carcinoma (PTC) accounts for approximately 84% of all cases, while anaplastic thyroid carcinoma (ATC) and poorly differentiated thyroid carcinoma (PDTC) account for less than 6% of cases, yet they cause nearly 80% of TC-related deaths [2]. This significant difference indicates that the molecular evolution characteristics of different TC subtypes vary greatly. Conventional therapies, including surgery and radioactive iodine (RAI) therapy, are ineffective for radioiodine-resistant differentiated thyroid carcinoma (RAIR-DTC), medullary thyroid carcinoma (MTC), ATC, and advanced or metastatic disease [3] (Fig. 1). Clinical data show that the median overall survival of patients with advanced anaplastic thyroid cancerATC is usually less than eight months [4]. This highlights the need to develop new treatment strategies.

Research focus has turned to the regulatory mechanisms of thyroid cancer itself, especially metabolic reprogramming and epigenetic remodeling. These mechanisms synergistically drive tumor development. The key epigenetic change—abnormal DNA methylation—usually manifests as local hypermethylation of CpG islands, accompanied by genome-wide hypomethylation [5]. This mechanism not only promotes genome instability but also directly silences key thyroid function genes (such as TSHR and NIS) and tumor suppressor genes (such as PTEN and RASSF1A), thus promoting the malignant progression of thyroid cancer. At the same time, metabolic reprogramming confers extraordinary adaptability to thyroid cancer cells.

For instance, the overexpression of GLUT1 can enhance the Warburg effect in differentiated thyroid carcinoma by upregulating LDHA [6]. This process acidifies the tumor microenvironment and promotes immune evasion. Furthermore, in RAIR-DTC, the resistance to radioactive iodine is caused by glycolysis triggered by the PI3K/AKT/mTOR pathway, and this glycolysis reduces the level of reactive oxygen species [7]. It is particularly important to note that an increasing number of studies have shown that in TC, there is a bidirectional relationship between DNA methylation and metabolic reprogramming. On one hand, metabolites (such as S-adenosylmethionine, abbreviated as SAM, and α-ketoglutarate) and related signaling pathways (such as PI3K/AKT and MAPK) dynamically regulate the activity of DNA methyltransferases, thereby enhancing the silencing effect mediated by DNA methylation on genes such as RASSF1A and promoting the formation of an invasive phenotype [8, 9]. However, by inhibiting the genes encoding metabolic enzymes (including HK2, FASN, and GLS1) as well as thyroid differentiation markers (such as NIS and TSHR), DNA methylation systematically reprograms the metabolic network of TC [10, 11]. This continuous interaction forms a self-reinforcing cycle, accelerating the development of cancer.

Based on this, this study innovatively explores the interactive pathways between DNA methylation and metabolic reprogramming in thyroid cancer. It constructs a multi-target therapeutic theoretical framework with the “DNA methylation-metabolism axis” as the core. By elucidating the tumor immune microenvironment, this framework not only significantly deepens the understanding of the development process of thyroid cancer but also provides a new perspective and transformative application path for precision medicine models, such as combined immunotherapy.

## Literature search strategy

The literature search method used in this study utilized the “ScienceNet Core Library” and “PubMed” databases. The search strategy was based on three core concepts: thyroid cancer, DNA methylation, and metabolic reprogramming. Search keywords and combinations include: “thyroid cancer”, “DNA methylation”, “metabolic reprogramming”, “Warburg effect”, “lipid metabolism” and “amino acid metabolism”. We will give priority to searching the English peer-reviewed literature for studies on the correlation between DNA methylation and metabolic reprogramming in thyroid cancer. Compound search queries are constructed through a combination of Boolean operators, focusing on original research and high-quality review literature published up to 2026. After excluding non-English literature, conference papers, editorials, letters, case reports, and studies with weak relevance to the topic, the inclusion criteria prioritize mechanism exploration and functional validation. After a preliminary screening of titles and abstracts and a review of the full texts, a total of 157 representative and methodologically reliable articles were finally included, ensuring that the selected review materials are comprehensive, timely, and have undergone strict evaluation.

## Dna Methylation: Fundamental Mechanisms and Biological Functions

DNA methylation is a core epigenetic mechanism regulating transcriptional plasticity and genome structure. This mechanism involves finely regulated systems, including DNA methyltransferases (DNMTs), ten-eleven translocation (TET) enzymes, and methyl-CpG binding proteins (MeCPs) [12]. This ternary regulatory axis coordinates key processes such as transcriptional regulation, genomic imprinting, X chromosome inactivation, and transposon silencing.

When DNA methyltransferases (DNMTs) use S-adenosylmethionine (SAM) as a methyl donor to transfer a methyl group to the C5 position of cytosine, the DNA methylation cycle is initiated. This process mainly occurs within CpG dinucleotides [13]. DNMT1 maintains the original methylation pattern during DNA replication, while DNMT3A and DNMT3B are responsible for the methylation process of nascent DNA. Conversely, TET enzymes oxidize 5-methylcytosine (5mC) sequentially into 5-hydroxymethylcytosine (5hmC), 5-formylcytosine (5fC), and 5-carboxylcytosine (5caC). Subsequently, the intermediate product 5caC is removed by thymine DNA glycosylase (TDG) and repaired through the base excision repair (BER) pathway, thereby achieving active demethylation and maintaining dynamic balance [9]. Subsequently, methyl-binding proteins (such as MeCP1, MeCP2) recognize and bind to methylation sites, recruit chromatin remodeling factors to interpret methylation patterns, and regulate gene accessibility [14]. Disruption of this balanced network is a hallmark feature of cancer development. In thyroid cancer (TC), this manifests as a classic “DNA methylation paradox”: global hypomethylation coexists with focal hypermethylation in the promoter regions of specific tumor suppressor genes [12]. During the malignant development of thyroid cancer, the reprogramming of DNA methylation is driven by multiple pathways. Abnormal DNMT activity is the main driving factor, for example, DNMT3B overexpression directly induces high methylation of promoters and inhibits tumor suppressor genes [15]. Additionally, DNA methylation interacts synergistically or antagonistically with histone modifications (such as H3K27ac) to finely regulate gene expression [16]. Although the dysfunction of the BER pathway can lead to abnormal accumulation of 5hmC and impaired demethylation [17], and in other cancers, non-coding RNAs have been confirmed to be important epigenetic regulators [18], their specific roles in TC still need to be further verified and explored.

**Fig. 2:**
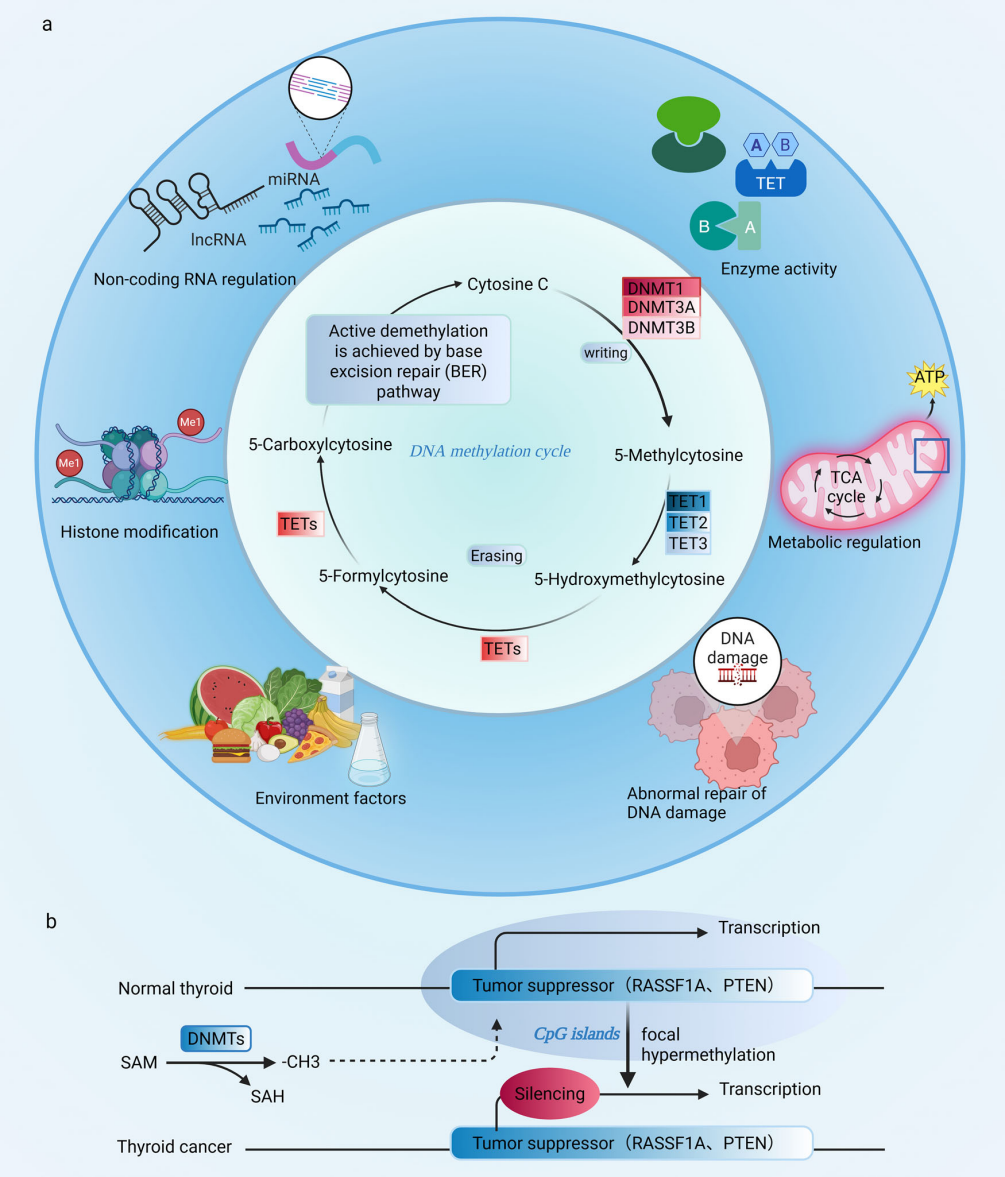
Conceptual diagram illustrating the role of DNA methylation in thyroid cancer (TC). In TC, DNA methyltransferases (DNMTs) catalyze the formation of 5-methylcytosine (5mC), while TET proteins use α-glycine as a cofactor to oxidize 5mC, thereby promoting the gradual demethylation process. The TDG/BER path supports this process. Environmental factors such as radiation and DNA damage response dysfunction can disrupt methylation dynamics. The promoter regions of CpG islands in tumor suppressor genes are highly methylated, leading to transcriptional silencing and promoting malignant transformation. Data Source: [9, 12–14]. Created with BioRender.com.

In summary, this dynamic regulatory network disorder is the significance mechanism of abnormal DNA methylation and malignant progression in TC (Fig. 2).

## Abnormal DNA methylation patterns in thyroid cancer

Abnormal DNA methylation is an important epigenetic driving factor in the formation of thyroid cancer (TC). It mainly regulates complex molecular regulatory networks through hypermethylation in the promoter regions of tumor suppressor genes and thyroid differentiation-related genes (such as TSHR and NIS). This abnormal mechanism promotes malignant transformation through dual effects: on the one hand, it simultaneously inhibits key tumor suppressor genes, and on the other hand, it activates oncogenic signaling pathways, ultimately leading to genomic instability.

### Bidirectional regulation of abnormal DNA methylation: from tumor suppressor gene silencing to oncogene activation

Abnormal DNA methylation plays a key role in the malignant development of TC through epigenetic regulatory mechanisms that both suppress tumor suppressor genes and activate oncogenes. This regulatory network is mainly manifested by abnormal hypermethylation of CpG islands in the promoter region, leading to transcriptional silencing of important tumor suppressor genes such as PTEN, RASSF1A, p16 (CDKN2A), and hMLH1. This destructive effect promotes genetic instability, disrupts cell signaling and cell cycle regulation, and jointly promotes metabolic reprogramming and the development of thyroid cancer. Specifically, this network functions at multiple levels to eliminate the limitations on cell survival, mobility, and proliferation. For example, in early thyroid cancer, hypermethylation of the promoter region inhibits the key PI3K/AKT pathway inhibitor PTEN. This process is mediated by activated DNA methyltransferases (DNMTs) driven by PI3K-AKT signaling, thereby establishing a self-reinforcing oncogenic cycle [19–21]. Similarly, hypermethylation of the RASSF1A gene (mainly found in BRAF-negative thyroid cancer) will disrupt its normal regulation of the Ras-MAPK signaling pathway, leading to continued activation of the cell cycle [22–24]. Hypermethylation of the key cell cycle inhibitor p16 (CDKN2A) simultaneously relieves the inhibition of the core proliferation pathways Ras-Raf-MEK-ERK and PI3K-AKT [25]. In BRAF V600E mutant papillary thyroid cancer, abnormal hypermethylation causes HOXD10 to lose its tumor suppressor function. This promotes tumor invasion by dysregulating pathways that normally suppress cell migration and induce apoptosis (such as RHOC/AKT/MAPK) [26, 27]. In addition to regulating proliferation and invasion, DNA methylation also supports cancer cell growth by reshaping stress responses and cellular metabolism. For example, hypermethylation can silence the important tumor suppressor gene transcription factor ATF3. Its deletion not only aggravates the dysregulation of key genes in the MAPK and PI3K/AKT pathways, but also directly interferes with the cell cycle and metabolic balance [28]. In addition, the estrogen-ERα/DNMT3B axis can induce gene hypermethylation, inhibit FAM111B gene expression, and directly promote the glycolysis process and metastasis ability of tumor cells [29, 30]. These findings highlight the direct impact of DNA methylation on metabolic phenotypes. Furthermore, this epigenetic network accelerates the evolution of tumor heterogeneity by promoting genetic instability. For example, hypermethylation of the mismatch repair gene hMLH1 synergizes with the BRAF V600E mutation to significantly increase the genomic mutation burden and promote the emergence of more aggressive and metabolically adaptable tumor clones [31]. Notably, aberrant DNA methylation not only promotes tumor progression by silencing tumor suppressor genes, but also achieves this goal by assisting in the activation of proto-oncogenes. In the absence of a TERT promoter mutation, hypermethylation of its upstream regulatory region will abnormally increase TERT transcription. The mechanism lies in recruiting the chromatin remodeling protein CHD4 and reducing repressive histone marks (such as H3K9me3/H3K27me3) [32]. In addition, studies have confirmed that hypomethylation of the promoters of genes such as PLAU can enhance their expression in differentiated thyroid cancer, thus constituting another risk factor for tumor recurrence [33]. In summary, bidirectional DNA methylation regulation outlines the genome-wide epigenetic landscape of malignant progression of thyroid cancer, covering the inactivation of tumor suppressor genes (such as PTEN, RASSF1A) and the abnormal activation of oncogenes (such as TERT, PLAU). These multilayered pathways illustrate how epigenetic dysregulation drives tumor progression through disrupted signaling, metabolic remodeling, and accelerated evolution. They laid a solid molecular foundation for the core concept of the “DNA methylation-metabolism axis”, thereby creating new opportunities for the development of precise therapies targeting this axis.

### Methylation-mediated thyroid-specific gene silencing

TC dedifferentiation and resistance to radioactive iodine (RAI) are primarily driven by the loss of activity in thyroid-specific genes, with DNA methylation-mediated transcriptional silencing being a key factor. Hypermethylation of critical gene promoters, such as NIS and TSHR, is strongly linked to a lower differentiation status and poor response to RAI therapy. The underlying molecular pathways have been partially identified. In vitro investigations have demonstrated that the aberrant activation of the MAPK signaling pathway in BRAF V600E-mutant TC can upregulate the production of DNMT1, thereby inducing hypermethylation of the TSHR promoter and leading to its transcriptional suppression [34, 35]. Impaired TSHR expression disrupts normal thyroid hormone signaling and directly reduces RAI uptake. Furthermore, the absence of differentiation markers such as thyroid globulin is positively correlated with the methylation level of TSHR, indicating that it has the ability to serve as a dynamic epigenetic biomarker for tracking dedifferentiation [36]. Similarly, the highly methylated CpG island in the NIS promoter significantly inhibits its transcription and reduces its localization on the membrane, thereby directly hindering the accumulation of iodine within the cells [36, 37]. Clinical evidence further demonstrates that the methylation level of NIS in tumor tissues is significantly negatively correlated with the iodine uptake rate of patients receiving radioactive iodine treatment [38, 39], clearly indicating that the methylation-induced silencing of NIS is the key direct cause of radioiodine resistance.

In summary, the methylation-mediated inactivation of thyroid-specific genes, including TSHR and NIS, is an important epigenetic mechanism leading to dedifferentiation and radioiodine resistance. Therefore, reversing this epigenetic silencing to restore gene expression and function provides an important therapeutic approach to overcome resistance.

## regulatory mechanisms of metabolic reprogramming on DNA methylation

The metabolic reprogramming in thyroid cancer (TC) not only maintains rapid growth by establishing a strong anabolic network, but also dynamically regulates the DNA methylation pattern. The key metabolites produced during this process maintain the Warburg effect [40, 41], and at the same time act as substrates, cofactors, or allosteric regulators for epigenetic enzymes (such as DNA methyltransferases (DNMTs) and ten-eleven translocases (TETs), thereby directly regulating their activity [8]. This change in metabolite abundance directly disrupts the balance of DNA methylation. Metabolic reprogramming and epigenetic remodeling thus jointly form a dual adaptation mechanism that drives malignant progression. Moreover, the key oncogenic signaling pathways in TC (such as PI3K-AKT, MAPK, Wnt/β-catenin, and HIF-1) can indirectly affect the activity of DNMT and TET by regulating the levels of key metabolic intermediates (such as acetyl coenzyme A and α-glutamine) [42]. This makes metabolic reprogramming a key link connecting abnormal upstream signals to downstream epigenetic reprogramming. In summary, these changes in the genome-wide methylation patterns triggered by metabolic processes help tumor cells invade, metastasize, and proliferate.

### Key features of metabolic reprogramming in thyroid cancer

#### Glucose metabolism reprogramming

Reprogramming of glucose metabolism is a key factor driving the malignant progression of thyroid cancer, providing energy and biosynthetic precursors for rapid tumor growth. This reprogramming is initiated by oncogenic signaling pathways. For example, the BRAF V600E mutation activates the MAPK/ERK pathway, inducing the expression of key glycolytic enzymes (including hexokinase 2 (HK2), 6-phosphofructokinase/fructose-2,6-diphosphatase 3 (PFKFB3), and lactate dehydrogenase A (LDHA)), and promoting the membrane localization of glucose transporter 1 (GLUT1), thereby establishing a continuous energy supply axis to provide power for tumor growth [37, 43]. Overexpression of these enzymes is closely associated with radioactive iodine (RAI) resistance and dedifferentiation, suggesting that they are potential metabolic biomarkers [44]. At the same time, the PI3K/AKT/mTORC1 pathway activated by G6PD activates the pentose phosphate pathway (PPP), forming a positive feedback loop between NADPH/GSH redox balance and mTOR lysosomal localization, further enhancing metabolic adaptability and survivability [6, 45]. In poorly differentiated tumor cells, the TCA cycle and pyruvate metabolic activities are significantly enhanced, and key metabolic nodes such as ATP-citrate lyase (ACLY) and lactate dehydrogenase A (LDHA) are finely regulated by transcription factors such as KLF12, ZNF281, and RELA through super enhancers. Inhibition of these factors disrupts tumor cell growth and energy balance [46–49]. In addition, the lactate dehydrogenase/M2-type pyruvate kinase (PKM2) molecular switch drives the lactate-acetyl-CoA axis, promotes histone H3K9 acetylation, and then activates epithelial-mesenchymal transition (EMT)-related transcription factors, demonstrating the role of metabolite-epigenetic interactions in tumor plasticity [43, 50]. Hypoxic signaling in the tumor microenvironment is mediated through HIF-1α and interacts with the above pathways, further strengthening the Warburg effect and enhancing invasiveness [51, 52].

In summary, glucose metabolism reprogramming in tumor cells is manifested by changes in the expression of metabolic enzymes, accumulation of by-products such as lactate, and changes in redox status, forming a dynamic network coordinated by multiple signaling pathways. The specific metabolites and microenvironment generated by this network provide a direct material basis and upstream signals for the subsequent abnormal regulation of DNA methylation patterns.

#### Lipid metabolism reprogramming

Reprogramming of lipid metabolism is a key feature in the development of TC by coordinating the synthesis and breakdown of fatty acids to meet the energy needs and biosynthetic requirements of the tumor. Dysregulation of fatty acid synthesis is primarily driven by oncogenic signals. Abnormal activation of the PI3K/AKT/mTOR-SREBP1c pathway up-regulates key lipid production genes, including fatty acid transporter (FATP1/2), acetyl-CoA carboxylase (ACC), fatty acid synthase (FASN) and stearoyl-CoA desaturase 1 (SCD1), thereby promoting the synthesis, invasion and transfer of new lipids [53]. In particular, SCD1, as an oncogene, is crucial for ATC cell survival, and its inhibition can trigger endoplasmic reticulum stress and apoptosis [54]. Similarly, pyruvate carboxylase (PC) enhances FASN expression and fatty acid synthesis by activating the AKT/mTOR/SREBP1c pathway, thereby accelerating tumor growth [47]. These findings indicate that SCD1 and FASN are potential therapeutic targets. Lipid oxidation (FAO) underlies metabolic adaptation and drug resistance. The BRAF V600E mutation promotes mitochondrial fatty acid oxidation by activating the ERK/ACC2 phosphorylation axis and releasing the inhibition of carnitine palmitoyltransferase 1 A (CPT1A) [55, 56]. After treatment with BRAF inhibitors, the AMPK-mediated feedback mechanism enhances ACC1 expression and lipid synthesis, revealing a metabolic adaptation mechanism that supports treatment resistance [57, 58]. In addition, hypoxia in the tumor microenvironment promotes CPT1A transcription through HIF-1α, enhancing fatty acid oxidation to maintain redox balance and promote survival [59, 60]. Lipid metabolism reprogramming is of great clinical significance in thyroid cancer. Overexpression of LPL, FATP2, and CPT1A can enhance the migration ability of PTC cells and is associated with poor prognosis, lymph node metastasis, and TNM stage progression [55]. Prediction models containing lipid metabolism genes (such as FABP4, PPARGC1A) can effectively stratify patient survival risks, and high-risk characteristics are associated with shortened survival and reduced immune infiltration [61]. In summary, lipid metabolism reprogramming in TC not only promotes malignant growth, but is also closely related to prognosis, immune infiltration, and treatment response. Targeting key nodes such as SCD1 or CPT1A has therapeutic potential, but further research is needed to overcome the limitations of current inhibitor therapies.

#### Amino acid metabolic reprogramming

Reprogramming of amino acid metabolism plays a key role in the malignant growth of thyroid cancer, in which the metabolic pathways of glutamine, serine, and proline are particularly important. First, by restructuring the amino acid transport network, cancer cells gain a metabolic advantage. The transport axis with LAT1/ASCT2/xCT as the core can form a “metabolic adsorption effect” to achieve efficient uptake of exogenous amino acids. Single-cell sequencing analysis showed that LAT1-overexpressing cells were enriched at the tumor invasion front, and their expression levels were closely related to lymph node metastasis [62]. At the functional level, the LAT1 inhibitor JPH203 inhibits the mTORC1-S6K signaling pathway by reducing leucine uptake. Its therapeutic potential is particularly prominent in the BRAFV600E/TP53 co-mutated mouse model—the inhibitor can downregulate CDK4/6 and cyclin D1 expression and achieve selective cytotoxicity [62]. The glutamine and serine metabolic pathways are critical for the metabolism of specific amino acids. A case-control study of patients with papillary TC showed that genetic variations in glutamine transporter genes SLC1A5/SLC1A3, serine metabolism gene SHMT1, and proline catabolism gene PRODH were significantly associated with disease risk. This suggests that changes in these genes may affect the intracellular amino acid metabolism network, ultimately leading to the formation of PTC [63]. Of particular importance is the direct functional relationship between serine metabolism and epigenetic regulation. Serine metabolism is closely linked to epigenetic regulation through a special de novo synthesis pathway triggered by PHGDH. According to isotope tracking studies, approximately 32% of labeled serine enters the single-carbon pool, providing the substrate SAM for DNA methylation, promoting the tumor genome to be in a highly methylated state [64].

In summary, through the reconstruction of the transmembrane transport network and the abnormal metabolism of key amino acids (including glutamine and serine), a metabolic network is formed that promotes the malignant development of thyroid cancer. Specifically, the metabolic flow allocated to the serine metabolic pathway during SAM synthesis clearly reveals how reprogramming of amino acid metabolism provides the material basis for DNA methylation, a key epigenetic change. This lays an important foundation for subsequent research on how metabolites affect epigenetic mechanisms through specific regulatory mechanisms.

## DNA methylation driven by metabolic reprogramming

**Fig. 3:**
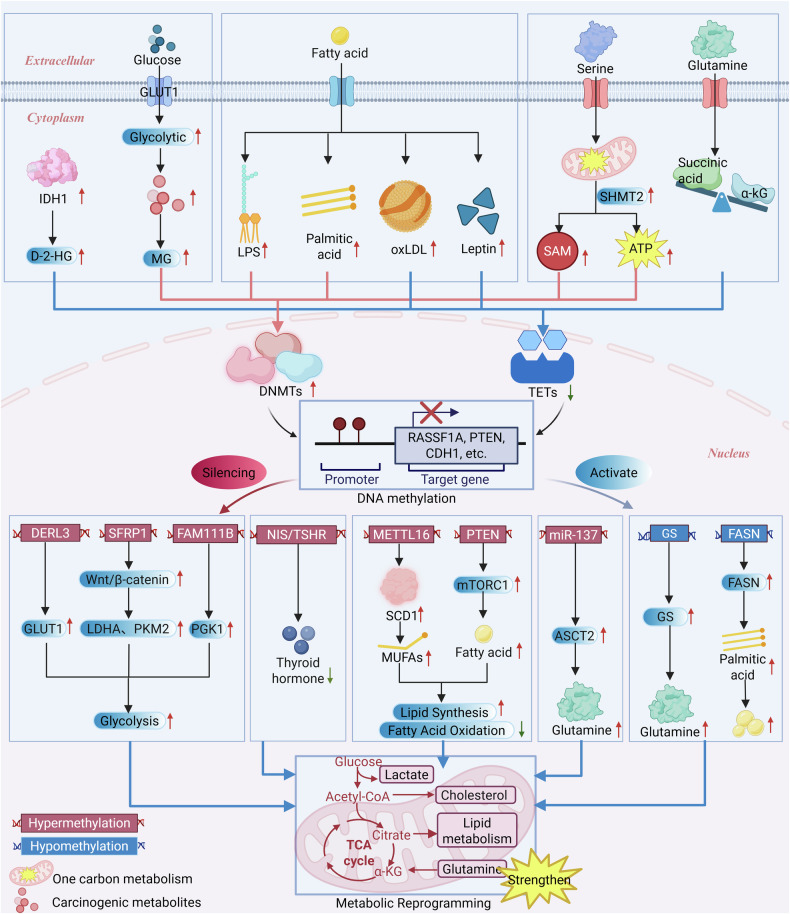
Conceptual diagram of metabolism-epigenetic interactions in thyroid cancer (TC). This schematic illustrates the bidirectional, self-reinforcing oncogenic cycle between metabolic reprogramming and DNA methylation in thyroid cancer. **Top:** Reprogramming of glucose, lipid, and amino acid metabolism changes the levels of key metabolites (e.g., SAM, α-KG, D-2-HG) that regulate the activity of DNMT and TET, thereby inducing hypermethylation in the promoter regions of tumor suppressor genes (e.g., RASSF1A, PTEN). **Bottom:** The resulting abnormal DNA methylation silences or activates specific genes (such as FAM111B, METTL16, FASN), thereby regulating signaling pathways (such as Wnt/β-catenin, mTORC1), and systemically enhancing tumor-promoting metabolic phenotypes, including glycolysis, lipid synthesis, and glutamine metabolism. This interactive cycle jointly promotes TC development and treatment resistance. Red and green arrows represent up-regulation/promotion and down-regulation/inhibition, respectively. **Data Source:**[20, 21, 30, 35, 55, 66–69, 71–90, 92, 93]. Created with BioRender.com.

Metabolic reprogramming not only provides energy and material basis for thyroid cancer (TC), but also serves as a key upstream event leading to abnormal DNA methylation, regulating cancer cell proliferation, differentiation, metastasis, and survival. By altering the metabolism of glucose, lipids, and amino acids, tumor cells adjust the intracellular levels of key metabolites (such as SAM, α-KG, and D-2-hydroxyglutarate (D-2-HG)). These metabolites directly regulate the activity and specificity of DNA methyltransferases (DNMTs) and ten-eleven translocation (TETs) enzymes through their roles as substrates, cofactors, or competitive inhibitors. Through this mechanism, adaptive metabolic states are “fixed” into stable epigenetic imprints. This section will systematically explain the “metabolic-epigenetic axis”, which is driven by metabolic reprogramming. This mechanism is crucial for understanding the malignant biological behavior of TC and developing new targeted therapeutic approaches **(**Fig. 3, Table 1).

**Table 1:** Core interactive mechanisms of DNA methylation and metabolic reprogramming in thyroid cancer (TC).

Regulatory Direction	Regulatory Type	Core Mechanism	Effect	Functional Significance	Level of Evidence	References
Metabolic Reprogramming → Regulation of DNA Methylation	Amino Acid Metabolism	Serine-One Carbon Metabolism (SHMT2 Overexpression) → Provides SAM (methyl donor) and ATP	Promotes DNA hypermethylation; ATP may synergistically enhance DNMT1 activity	SHMT2 overexpression drives metastasis and promotes an invasive phenotype	In vitro + Animal Studies	[21, 75–77]
		Glutamine Metabolism → Generates α-KG (TET cofactor)	Changes in α-KG concentration affect the efficiency of TET-mediated demethylation	Restoring α-KG can inhibit tumor growth	In vitro Studies	[78, 79]
DNA Methylation → Driving Metabolic Reprogramming	Regulation of Glucose Metabolism	DNMT3B-mediated FAM111B Promoter Hypermethylation → Silencing of FAM111B	Relieves inhibition of glycolysis, upregulates glycolytic genes (e.g., PGK1)	Directly drives glycolytic reprogramming, promoting growth and metastasis	In vitro + Animal Studies	[30]
		DERL3 Promoter Hypermethylation → Silencing of DERL3	Relieves its transcriptional repression of GLUT1, exacerbating glycolysis	Forms a “DNA methylation-glucose transport-metabolic flux” positive feedback loop	In vitro Studies	[80]
		NIS/TSHR Promoter Hypermethylation → Silencing of thyroid function genes	Disrupts thyroid hormone synthesis and signaling, indirectly affecting glucose-lipid metabolic homeostasis	Reinforces the Warburg effect in the tumor microenvironment, leading to RAI resistance	In vitro + Animal Studies	[35, 84]
	Regulation of Lipid Metabolism	METTL16 Promoter Hypermethylation → Inhibition of its m⁶A modification function	Leads to SCD1 protein accumulation, promoting monounsaturated fatty acid (MUFA) generation	Enhances lipid metabolic reprogramming, promoting tumorigenesis	In vitro + Animal Studies	[85]
		FASN Promoter Hypomethylation	Enhances FASN transcription, driving de novo lipid synthesis	Supports proliferative demands (e.g., biomembrane synthesis), may be associated with therapy resistance	In vitro Studies	[10, 86–88]
		PTEN Promoter Hypermethylation → Silencing of PTEN → Activation of mTORC1	Promotes fatty acid uptake, inhibits β-oxidation, causing “mitochondrial metabolic reprogramming”	Forces cells to rely more on glycolysis, supporting malignant progression	Cohort Study	[20, 21]
	Regulation of Amino Acid Metabolism	Myc/TDG/TET3-mediated Active Demethylation of the glutamine synthetase (GS) Promoter	Relieves inhibition, upregulates GS expression	Provides endogenous glutamine for the tumor, potentially promoting malignant progression	In vitro Studies	[92]
		miR-137 Promoter Hypermethylation → Silencing of miR-137	Relieves its inhibition of the glutamine transporter ASCT2, enhancing glutamine uptake	Reprograms the amino acid metabolic network, supporting rapid proliferation	In vitro Studies	[93]

### Regulation of DNA methylation by glucose metabolism

Although traditional views emphasize the one-way regulatory effect of DNA methylation on metabolism [65], recent studies have shown that in thyroid cancer, the reprogramming of glycolysis may instead drive abnormal DNA methylation, providing a new theoretical perspective for targeted intervention (Fig. 3). Currently, the global regulatory mechanism is still not fully clear. However, two possible pathways regulated by specific metabolites have begun to emerge.

Specifically, changes in the key metabolic enzyme IDH1 in the TCA cycle have been found in TC [66]. Although the downstream carcinogenic mechanism of this specific cancer type is still unclear, studies on other cancers have revealed clear mechanism patterns. The carcinogenic metabolite D-2-HG competitively inhibits the function of TET peroxidase, and due to IDH1 mutations, it accumulates abnormally in glioma [67]. This leads to a high DNA methylation phenotype throughout the genome, thereby promoting cancer development [68, 69]. This conserved mechanism in gliomas and cholangiocarcinomas provides a rationale for the hypothesis that a similar ‘D-2-HG–TET inhibition’ axis may contribute to DNA hypermethylation in the rare subset of thyroid carcinomas harboring IDH1 mutations [68]. In addition to D-2-HG, other glucose metabolism byproducts have also been found to influence DNA methylation directly. For instance, through DNMT3B, methylglyoxal (MG), a byproduct of glycolysis, can cause hypermethylation of tumor suppressor genes in breast cancer [55], demonstrating that metabolites act as “epigenetic messengers.” It is unclear, nevertheless, whether MG controls DNMTs in TC (especially the BRAF V600E mutant subtype) via a comparable mechanism, causing essential tumor suppressor genes, such as RASSF1A or PTEN, to become hypermethylated and promoting dedifferentiation and treatment resistance. To functionally validate this theory, future research may integrate metabolomics with epigenetic editing tools, such as CRISPR-dCas9-TET1 [56, 70]. Furthermore, whether other glucose metabolites (such as lactate or intermediate metabolites) feedback-regulate DNA methylation by influencing methyltransferase activity or substrate availability remains to be studied. These studies will reveal novel metabolic-epigenetic dual targets for the treatment of TC and deepen our understanding of the role of glucose metabolism reprogramming in epigenetics.

### Regulation of DNA methylation by lipid metabolism

In thyroid cancer, lipid metabolism reprogramming not only supports biosynthesis and energy supply, but may also directly induce aberrant DNA methylation through the production of key metabolites. These metabolites function as regulators of epigenetic enzymes, thereby establishing a critical link between metabolism and epigenetics **(**Fig. 3**)**. Although direct evidence in TC remains limited, studies in other disease models have provided valuable insights. First, the DNA demethylation process can be regulated by lipid metabolites. For example, oxidized low-density lipoprotein (oxLDL) can significantly reduce the expression of the key demethylase TET2 in trophoblast cells, leading to changes in DNA methylation patterns [71]. Secondly, the expression and activity of DNMTs are also affected by lipid metabolites. In hepatic steatosis models, lipopolysaccharide (LPS) induces hypomethylation of the CIDEA promoter and activates its transcription by inhibiting DNMT3B expression [72]; studies on type 2 diabetes have found that palmitic acid activates the AMPK signaling pathway and promotes the transport of DNMT1 to mitochondria, resulting in hypermethylation of the mitochondrial gene ND6 [73]. These findings indicate that lipid metabolites can have different regulatory effects on DNA methylation in nuclear and mitochondrial genomes. Furthermore, there is a dynamic bidirectional relationship between metabolites and epigenetic systems. In energy metabolism research, TET2 enhances leptin gene expression by binding to the transcription factor C/EBPα; conversely, high leptin levels inhibit TET2 expression through the JAK2-STAT3 signaling pathway, forming a “TET2-leptin” negative feedback loop [74]. This suggests that metabolites are both positive regulators and passive targets of epigenetic regulation. Based on the regulatory pathways established in various physiological and pathological conditions, we propose the following hypothesis: The lipid metabolism reprogramming that occurs in the tumor microenvironment of TC may similarly disrupt the methylation patterns of specific gene regions (such as the promoter regions of tumor suppressor genes) by interfering with the demethylation process mediated by TET proteins and the methylation process mediated by DNA methyltransferases (DNMTs). This theory provides a fresh viewpoint on the relationship between metabolism and epigenetics in TC. However, additional experimental validation is needed for the particular metabolites implicated, their target molecules, and the upstream and downstream pathways in TC.

### Regulation of DNA methylation by amino acid metabolism

By controlling the amounts of essential metabolites SAM and α-KG, the reprogramming of amino acid metabolism in TC dynamically affects the stability of DNA methylation states. The synergistic control of methyl donor supply and demethylase activity is the primary focus of this process **(**Fig. 3**)**. First, the serine-one-carbon metabolic pathway provides the required substrates and energetic support for DNA methylation. This metabolic pathway yields the one-carbon unit necessary for the formation of the methyl donor SAM through processes mediated by serine hydroxymethyltransferase 2 (SHMT2) [75]. SHMT2 is overexpressed in TC tissues and has a favorable correlation with CpG island hypermethylation and lymph node metastatic risk, according to clinical pathological investigation [21, 76]. In addition to providing methyl donors, serine metabolism maintains high intracellular ATP levels by contributing to the synthesis of purine nucleotides. Studies have found that ATP can enhance the localization and catalytic activity of DNMT1 in the nucleus [77]. This raises a key scientific question: Does SHMT2 overexpression increase SAM levels and maintain cellular high-energy status by synergistically enhancing DNMT1 activity? Could this mechanism ultimately lead to an aggressive phenotype by promoting silencing of anti-metastasis genes such as CDH1? This hypothesis can be tested by interfering with SHMT2 and the one-carbon metabolism pathway, while monitoring intracellular ATP levels, DNMT1 activity and the methylation status of specific genes. In contrast, α-KG is an essential cofactor for TET peroxidase, and changes in its concentration can directly regulate DNA demethylation effects. Functional tests have shown that the glutaminase inhibitor CB-839 can limit α-KG synthesis, thereby reducing TET enzyme activity and significantly inhibiting TC cell proliferation [78, 79].

In summary, by selectively regulating key metabolites such as SAM and α-KG, amino acid metabolism reprogramming of tumor cells systematically regulates DNA methylation patterns through multiple mechanisms. These mechanisms include methyl donor supply, demethylase activity, and energy balance regulation, providing new molecular understanding and intervention possibilities for anti-cancer strategies focusing on the “metabolism-epigenetic axis”.

## DNA methylation-driven metabolic reprogramming

The previous article elaborated on how metabolic reprogramming establishes an epigenetic environment that enables epigenetic reprogramming by providing key metabolites that regulate DNA methylation. This section examines the inverse relationship: how DNA methylation itself acts as a stable upstream regulator that directly influences metabolic reprogramming in thyroid cancer. DNA methylation systematically restructures cellular metabolic networks by silencing or activating specific sets of genes, including markers of thyroid differentiation (e.g., TSHR, NIS) and key metabolic enzymes in glucose, lipid, and amino acid pathways. This reprogramming drives the development of malignant phenotypes such as invasion, metastasis, and proliferation, thereby consolidating the self-reinforcing properties of the methylation-metabolism axis (Fig. 3, Table 1).

### Regulation of glucose metabolism by DNA methylation

In TC, DNA methylation profoundly reshapes glucose metabolism through a multi-level regulatory network, which not only directly epigenetically regulates metabolic genes, but also indirectly affects key signaling pathways (Fig. 3). First, it directly silences specific tumor suppressor genes to relieve inhibition of glycolysis. For example, estrogen signaling can upregulate the DNMT3B gene, leading to its hypermethylation and suppression of the tumor suppressor gene FAM111B. This defect enhances glycolysis (increases glucose uptake, lactate production, and extracellular acid release rate) and directly upregulates key glycolysis genes such as PGK1 [30], thereby establishing the DNMT3B/FAM111B/PGK1 axis as a key pathway for methylation-driven glycolysis reprogramming. Secondly, methylation affects metabolic characteristics by regulating glucose transport genes. In a variety of solid tumors, including thyroid cancer, promoter hypermethylation inhibits DERL3 and weakens its inhibitory effect on GLUT1, thus accelerating the expression of the glycolysis pathway and HK2 [80]. Together, these changes create a positive feedback loop that regulates the mutual regulation of DNA methylation and glucose transport and metabolism. Third, DNA methylation indirectly promotes metabolic reprogramming by inhibiting pathway inhibitors. For example, in various cancers such as colorectal cancer and breast cancer, promoter hypermethylation usually inhibits the Wnt/β-catenin antagonist SFRP1, resulting in continued activation of this pathway [81, 82]. The activated Wnt/β-catenin pathway directly upregulates the expression of glycolytic genes such as PKM2 and LDHA, thereby promoting the Warburg effect [83]. Given that activation of the Wnt pathway and enhanced glycolysis are ubiquitous in thyroid cancer, we hypothesize that SFRP1 promoter hypermethylation may be a key mechanism link. This hypothesis requires experimental verification. Finally, aberrant methylation of thyroid-specific genes indirectly promotes TC development by disrupting the local metabolic environment. NIS promoter hypermethylation not only hinders iodine uptake, but also disrupts systemic glucose-lipid balance [84]; while TSHR promoter hypermethylation aggravates the local Warburg effect by interfering with hormone signaling, affecting insulin sensitivity and glycolytic enzyme activity [35]. In summary, DNA methylation is the core mechanism driving glucose metabolism reprogramming in thyroid cancer. In-depth analysis of this regulatory network will provide a key theoretical basis for the development of new therapies targeting DNA methyltransferases, which are designed to reverse the Warburg effect and break through treatment resistance.

### Regulation of lipid metabolism by DNA methylation

DNA methylation dynamically regulates the expression of lipid metabolism genes in TC and reshapes intracellular lipid synthesis and catabolism, thereby supporting the malignant growth of tumors (Fig. 3).

First, DNA methylation promotes lipid synthesis through multiple mechanisms. In PTC, DNMT1-mediated METTL16 promoter hypermethylation inhibits its m6A modification function on SCD1 mRNA and blocks YTHDC2-mediated RNA degradation, leading to SCD1 protein accumulation and overproduction of monounsaturated fatty acids (MUFAs) [85]. At the same time, hypomethylation of the FASN promoter in TC enhances its sensitivity to the transcription factor SREBP-1c—a mechanism that has been shown to stimulate palmitic acid production in other cancers [86–88], indicating that there is a conserved lipid synthesis driving mechanism in thyroid cancer. In terms of fatty acid oxidation, PTEN promoter hypermethylation activates the mTORC1 signaling pathway, triggering a dual metabolic shift: both inhibiting the expression of carnitine palmitoyltransferase 1 A (CPT1A) and inhibiting mitochondrial β-oxidation. This metabolic shift forces cells to rely more on glycolysis to support biosynthesis, while upregulating fatty acid transporters to enhance the uptake of exogenous fatty acids [20, 21]. Furthermore, methylation of thyroid-specific genes indirectly affects lipid metabolism. Hypermethylation of TSHR and NIS promoters reduces thyroid hormone levels and inhibits adipose tissue triglyceride lipase (ATGL) activity. This effect synergizes with the PPARγ signaling pathway activated by CD36-mediated fatty acid uptake to promote lipid droplet accumulation [89, 90]. It is worth noting that the DNA methylation pattern that regulates lipid metabolism through genes such as FASN and SREBF1 in metabolic diseases has clear pathological significance in TC [91]: disrupting this network (such as blocking FASN) can make TC cells sensitive to the BRAF inhibitor vemurafenib [10], directly revealing the connection between epigenetic regulation and treatment resistance.

In summary, lipid metabolism in TC is regulated by plastic epigenetic networks. Future studies should elucidate the subtype specificity of these regulatory circuits and their potential role in treatment resistance.

### Regulation of amino acid metabolism by DNA methylation

By precisely regulating the expression of key genes involved in amino acid metabolism, DNA methylation drives the metabolic reprogramming of TC, thus laying the genetic basis for their malignant growth and treatment resistance **(**Fig. 3**)**. In the core network of glutamine metabolism, DNA methylation exhibits multi-level network regulatory characteristics.

On the one hand, active DNA demethylation can directly trigger the expression of key synthetic enzymes. For example, the oncogene Myc relieves transcriptional repression in various cancers by upregulating its direct target gene thymine DNA glycosylase (TDG). Subsequently, the glutamine synthetase (GS) gene promoter undergoes active demethylation together with TET3 under the action of TDG, resulting in a significant increase in GS expression and the production of new glutamine [92]. If this process is initiated simultaneously in TC, it may provide endogenous glutamine to tumors, thereby accelerating disease progression and enhancing treatment resistance. On the other hand, high DNA methylation affects glutamine uptake and metabolic flux by regulating transporters and related signaling cascades. For example, when the promoter of miR-137 is highly methylated, the cell’s ability to uptake glutamine is enhanced, thereby reducing its inhibitory effect on the glutamine transporter ASCT2 [93]. Taken together, these epigenetic regulatory processes alter the amino acid metabolism network of TC cells.

Future research should systematically elucidate the dynamic regulatory network of amino acid metabolism during the progression of thyroid cancer, focusing on analyzing the functions of epigenetic key nodes such as glutathione S-transferase (GST) and microRNA-137 in the drug resistance and carcinogenesis process, and exploring the therapeutic potential of these nodes. These measures will provide new strategies for overcoming the metabolic vulnerabilities of thyroid cancer.

## Therapeutic potential and strategies targeting methylation-metabolism cross-regulation

The treatment approach for thyroid cancer (TC) is shifting from a single targeted therapy to a multi-dimensional combined strategy. Given the close bidirectional interaction between DNA methylation and metabolic reprogramming, targeted treatment targeting this interrelated network is a promising therapeutic avenue to overcome treatment resistance [36, 94]. Therefore, understanding the molecular mechanism of this network and precisely targeting its key nodes may lead to new strategies. To proceed with the discussion, the key actionable targets within this axis have been systematically summarized (Table 2), classified based on their roles in the network and their correlations with specific TC subtypes. This overview provides guidance for the detailed treatment strategies discussed in the subsequent chapters.

**Table 2:** Therapeutic targeting of key nodes in the DNA methylation-metabolism axis of thyroid cancer: a target-subtype grid.

Key Node	Target	Primary TC Subtype(s)	Mechanism / Effect of Targeting	Exemplary Drug / Inhibitor	References
Metabolic Reprogramming → Influencing Methylation	SHMT2 (One-carbon metabolism)	PTC (Aggressive/Metastatic)	Depletes SAM pool, disrupts DNMT1 activity, reverses hypermethylation	(Potential target, investigational inhibitors)	[21, 75–77, 150]
	Glutaminase (GLS1)	ATC, RAIR-DTC	Reduces α-KG, inhibits TET activity, may alter methylation landscape	CB-839	[78, 79]
DNA Methylation → Driving Metabolism	FAM111B promoter (silenced)	PTC	Re-expression inhibits glycolytic genes (e.g., PGK1), reverses Warburg effect	DNMT inhibitor (e.g., Decitabine)	[30]
	METTL16 promoter (silenced)	PTC	Restores m⁶A-mediated decay of SCD1 mRNA, inhibits lipogenesis	DNMT inhibitor (e.g., Decitabine)	[85]
	FASN promoter (active)	Multiple (PTC, FTC)	Inhibits de novo fatty acid synthesis, targets metabolic dependency	TVB-2640 (FASN inhibitor)	[10, 86–88]
Shared/Upstream Nodes	BRAF V600E (MAPK pathway)	PTC, ATC	Redifferentiation: re-express NIS/TSHR; Direct inhibition	Dabrafenib, Vemurafenib	[113, 114]
	DNMT1 (Epigenetic writer)	RAIR-DTC, ATC	Global demethylation, re-expression of silenced genes (NIS, TSHR, RASSF1A)	Decitabine, Azacitidine	[95, 96]
	PD-1/PD-L1 (Immune checkpoint)	ATC, PDTC	Reverses immunosuppressive microenvironment shaped by metabolism & methylation	Pembrolizumab	[132, 134]

### DNA methylation targeted therapy: from monotherapy to combination strategies

Abnormal DNA methylation significantly affects the growth and progression of thyroid cancer. The proportion of hypermethylated DNA in TC tissue is significantly higher than that in normal tissue (74%–80% vs. 6%) [95]. In addition, the B-RafV600E mutation can upregulate DNA methyltransferase 1 (DNMT1) through the NF-κB pathway, leading to hypermethylation and silencing of the NIS promoter [96]. These findings provide a strong basis for treatments targeting DNA methylation.

However, DNA methyltransferase inhibitors (DNMTi) alone have limited efficacy in inducing “redifferentiation.” A phase II study (NCT00085293) showed that DNMTi monotherapy failed to restore radioactive iodine (RAI) uptake. In colorectal and breast cancer models, the anti-cancer mechanism of DNMTi is mainly due to its ability to induce DNA damage, a process that relies on the presence of DNMT1 in tumor cells [97]. This suggests that demethylation therapy alone may not be sufficient to achieve significant efficacy. Azacitidine, another DNMT inhibitor, can improve genome stability by restoring the function of the mismatch repair gene hMLH1 [98]. Its clinical potential to restore radioactive iodine (RAI) uptake in metastatic or persistent TC is under investigation (NCT00004062), although data are not yet available.

To overcome the limitations of monotherapy, DNMTi holds considerable promise in combination regimens. Preclinical studies show that combining decitabine with histone deacetylase inhibitors (e.g., sodium butyrate) synergistically upregulates NIS mRNA expression (by up to 1600-fold) and significantly increases iodine uptake (9-fold in DRO cells, 8-fold in 2-7 cells) [95], supporting epigenetic combination therapy as a viable strategy to overcome resistance. Synergy is also evident when DNMTi are combined with pathway-targeted agents. In medullary thyroid carcinoma models, decitabine plus the mTOR inhibitor everolimus demonstrated strong synergistic antiproliferation (synergy index CI = 0.1) and apoptosis (90% increase in late apoptotic/necrotic cells), mediated by activation of the NGFR-MAPK10-TP53-Bax/Bcl-2 pathway [99]. Decitabine can also upregulate PTEN via demethylation, thereby inhibiting the PI3K/AKT/mTOR pathway in T-cell acute lymphoblastic leukemia (T-ALL) models [100]. Combined with MEK inhibitors, it concurrently suppresses MAPK signaling and reverses epigenetic silencing, yielding synergistic antitumor effects in breast cancer and melanoma [101–103]. This synergy effect may arise because pathway inhibitors can block the downstream survival signals, while DNA methyltransferase inhibitors (DNMTi) can restore the expression of tumor suppressor genes, making cells more sensitive to apoptosis. Low-dose decitabine has also been proven to enhance the efficacy of chimeric antigen receptor T-cell (CAR-T) therapy [104], further expanding its application scope in combination therapy.

The emerging epigenetic editing technologies, such as CRISPR-dCas9, are improving the precision in this field. This system can reversibly and precisely edit the methylation status of specific sites (such as RASSF1A, PTEN) by fusing dCas9 with DNMT3A or TET1 and combining it with optogenetic regulation [56]. Unlike the broad effects of traditional DNMT inhibitors, this technology is expected to achieve targeted intervention at key nodes of the “DNA methylation-metabolism axis”, thereby reducing systemic toxicity and overcoming drug resistance.

**Fig. 4:**
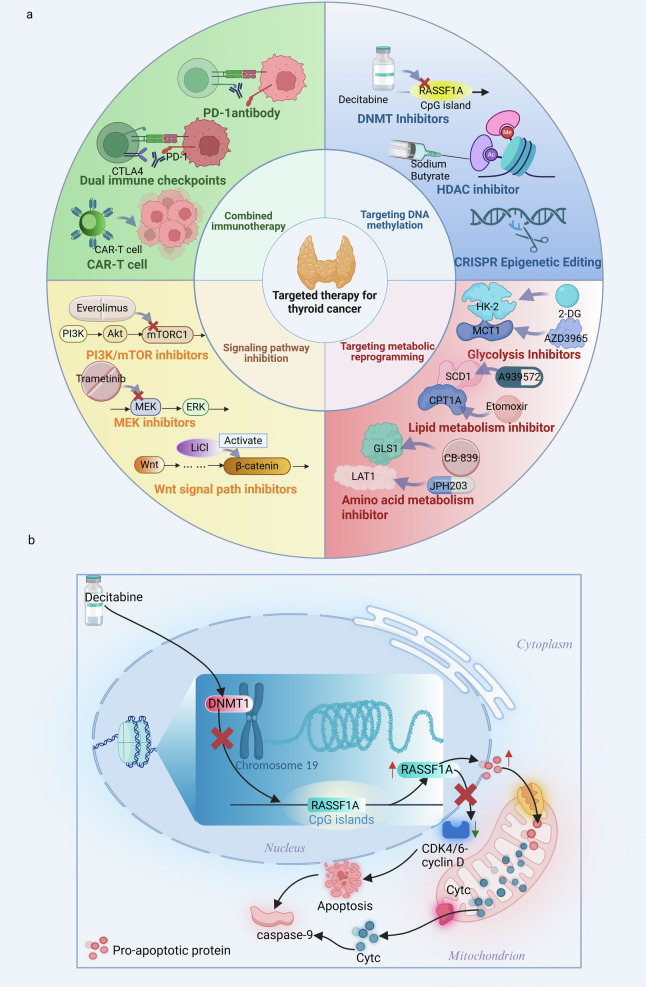
Conceptual schematic of an integrated therapeutic strategy targeting the epigenetic-metabolic axis in thyroid cancer (TC). **a** The integrated strategy synergistically combines epigenetic modulators, metabolic inhibitors, and signaling pathway blockers. Representative agents include DNMT inhibitors (e.g., decitabine), metabolic drugs targeting glycolysis, lipogenesis, and glutaminase (e.g., 2-DG, A939572, CB-839), and kinase inhibitors against BRAF, MEK, and mTOR (e.g., dabrafenib, trametinib, everolimus). These are often used together with immune checkpoint inhibitors such as pembrolizumab to remodel the tumor microenvironment. **b** DNA methyltransferase inhibitors (e.g., decitabine) act primarily by inhibiting DNMT1. This reverses aberrant promoter hypermethylation of key tumor suppressor and thyroid function genes, including RASSF1A, PTEN, and NIS, leading to their re-expression and resulting in cell cycle arrest and apoptosis. Combining them with HDAC inhibitors (e.g., sodium butyrate) significantly enhances this redifferentiation effect. Data Source: [20, 36, 94, 142, 156, 157]. Created with BioRender.com.

In summary, the targeted treatment mode for tumor DNA methylation has evolved from the early “re-differentiation based on a single therapy” model to the modern “multi-axis intervention” strategy. This new approach synergistically integrates epigenetic modulators, signaling pathway inhibitors, immunotherapies, and advanced epigenetic tools, offering a promising avenue to improve patient outcomes and overcome treatment resistance (Fig. 4).

### Metabolic intervention: exploring multi-level targeted strategies

Metabolic intervention in TC has evolved into a multi-tiered strategy targeting carbohydrate metabolism, lipid metabolism, and specific oncogenic mutations. Targeting carbohydrate metabolism, the hexokinase inhibitor 2-deoxyglucose (2-DG) suppresses lactate production and cell proliferation in PTC models [105]. In anaplastic TC, inhibiting the lactate exporter monocarboxylate transporter 1 (MCT1) with AZD3965 acidifies the tumor microenvironment and elevates apoptosis by 2.3-fold [106]. Although clinical application of AZD3965 is limited by toxicities such as retinopathy [107], its therapeutic window could be expanded through dose optimization or pH-activated nanodelivery systems [108, 109]. During the lipid metabolism process, the inhibitor A939572 of fatty acid acyl-CoA desaturase 1 (SCD1) exhibits strong anti-cancer activity in PTC cells (IC₅₀ = 820 nanomoles), and can reverse the tumor growth and lipid accumulation phenomena caused by METTL16 gene knockout [85]. Inhibition of fatty acid oxidation by carnitine palmitoyltransferase 1 A (CPT1A) inhibitors (such as etomidate) can also enhance chemotherapy sensitivity [110, 111]. In addition to the core pathway, correcting the pathogenic metabolism driven by specific mutations is also a more refined strategy. Although relatively rare in TC, the example established for IDH1 mutant glioma provides guidance. The mutant isocitrate dehydrogenase 1 (IDH1) inhibitor ivositinib can selectively reduce the levels of the oncogenic metabolite D-2-HG, thereby reversing the genome-wide DNA hypermethylation induced by it [112]. This suggests that precise metabolic regulation can repair downstream epigenetic dysfunction, providing a potential therapeutic rationale for treating similar driver mutations in aggressive TC.

### Signal pathway targeted therapy and redifferentiation strategies

For inhibitors targeting the MAPK pathway, such as BRAF and MEK inhibitors, they have dual mechanisms of action in the treatment of advanced TC: directly inhibiting tumor growth and restoring the affinity for radioactive iodine (RAI) through “redifferentiation”. Dabrafenib monotherapy induced new RAI uptake in 60% (6/10) of BRAF V600E mutant RAIR-PTC patients, while reducing serum thyroglobulin levels [113]. Dabrafenib combined with trametinib achieved an objective response rate of 69% in BRAF V600E mutant ATC patients, with a 12-month progression-free survival (PFS) rate and overall survival (OS) rate of 79% and 80%, respectively [114]. Similarly, the MEK inhibitor selumetinib enhanced iodine uptake in 60% (12/20) of RAIR-DTC patients, enabling 40% (8/20) of patients to receive RAI treatment, and reducing serum Tg by an average of 89% [115]. The mTOR inhibitor everolimus also showed broad activity. A phase II trial for RAIR-DTC reported a 58% disease control rate, with a median PFS of 9 months and OS of 18 months [116]. Pharmacokinetic analysis in this trial revealed a critical association between drug exposure and toxicity—higher drug exposure was associated with a higher incidence of oral ulcers (*P* = 0.047), and this was more pronounced in patients requiring dose reduction [117]—highlighting the importance of balancing efficacy and safety through therapeutic drug monitoring. In addition, developmental pathways such as Wnt/β-catenin have become new therapeutic targets. In FTC and ATC, AHNAK2 promotes the translocation of β-catenin to the nucleus and upregulates glycolysis genes (such as c-MYC, LDHA), thereby promoting metastasis. In contrast, the Wnt/β-catenin activator LiCl reversed all anti-tumor phenotypes induced by AHNAK2 gene silencing [118], indicating that inhibition of this pathway can delay TC progression.

In summary, signaling pathway inhibitors combat advanced TC through direct cytotoxic effects and epigenetic redifferentiation mechanisms. Its efficacy stems from the regulation of the “signaling-metabolism-epigenetic” interconnected network, such as reversing methylation-mediated gene silencing by down-regulating DNMT1. This provides a theoretical basis for designing a synergistic multi-target treatment plan (Table 3).

**Table 3:** Summary of thyroid cancer (TC) interventions and clinical trials.

Intervention/Drug		Study Model/Population	Sample Size	Control Group	Primary Outcome	Key Results	Level of Evidence	NCT Number	Refs
Epigenetic Drugs	Decitabine + I-131	RAI-refractory metastatic TC	12 patients	Self-controlled (before/after)	Restoration of RAI uptake	0/12 restored RAI uptake	Phase II (Human)	NCT00085293	[36, 151]
	Decitabine + Sodium Butyrate	Human TC cell lines (DRO, 2-7)	N/A	Normal thyroid tissue	Iodine uptake	Iodine uptake ↑9-fold (DRO); ↑8-fold (2-7)	Preclinical (In vitro)	N/A	[95]
	Azacitidine + I-131	Persistent/metastatic follicular/papillary TC	38 patients	Self-controlled	Restoration of RAI uptake	Results not reported	Phase I (Human)	NCT00004062	-
	Decitabine + Everolimus	Human MTC cell lines	N/A	Untreated/Monotherapy control	Synergistic antiproliferation (CI), apoptosis	CI = 0.1; Late apoptosis/necrosis +90%	Preclinical (In vitro)	N/A	[99]
Metabolic & Signaling Pathway-Targeted	AZD3965 (MCT1 inhibitor)	ATC cell lines (8505 C, JL30, TCO1)	3 cell lines	High-glucose medium	Cell proliferation	Suppressed proliferation in low-glucose (p ≤ 0.01)	Basic Research	N/A	[106]
	A939572 (SCD1 inhibitor)	PTC cell lines (BCPAP, K1, TPC1)	3 experiments	DMSO vehicle	Proliferation, clonogenicity, migration, TG	IC₅₀ = 820 nM; Inhibited proliferation & migration; reduced TG	Low (In vitro)	N/A	[85]
	A939572 (SCD1 inhibitor)	METTL16-knockdown nude mouse xenografts	n = 5/group	Vehicle control	Tumor volume/weight, SCD1, Ki-67	Reversed tumor growth & lipid accumulation	Medium (Animal)	N/A	[85]
Redifferentiation & Combination Therapy	Dabrafenib (BRAF inhibitor)	BRAF V600E-mutant RAIR-PTC	10 patients	Self-controlled	Proportion with new RAI uptake	60% (6/10) had new RAI uptake; 2 PR, 4 SD	Phase II Clinical Trial	NCT01534897	[113]
	Selumetinib (MEK inhibitor)	Advanced iodine-refractory TC	20 patients	None (single arm)	I-124 PET uptake increase	60% (12/20) had ↑I-124 uptake; 5/8 achieved PR after I-131	Phase II Clinical Trial	NCT00970359	[115]
	Dabrafenib + Trametinib	BRAF V600E-mutant ATC	16 patients	None (single arm)	Objective Response Rate	ORR 69%; 12-month PFS 79%, OS 80%	Phase II (Single arm)	NCT02034110	[114]
	Sorafenib + Everolimus	Advanced RAIR Hürthle cell carcinoma	35 patients	Sorafenib monotherapy	Progression-Free Survival	No PFS improvement; ORR 0%; ≥G3 AEs 88%	Phase II Randomized Trial	NCT02143726	[152]
	Selumetinib + RAI (Adjuvant)	High-risk DTC post-op	233 patients	Placebo + RAI	18-month Complete Response Rate	CR rate 40% vs 38% (NS); G3 AEs 16% vs 0%	Phase III RCT	NCT01843062	[153]
	Everolimus (mTOR inhibitor)	Advanced TC (DTC, ATC, MTC)	42 patients (PK analysis)	None (single arm)	Exposure-toxicity correlation (dose reduction, stomatitis)	Higher AUC in dose-reduced pts; stomatitis linked to exposure; ABCB1 genotype affected exposure	Phase II Clinical Trial	NCT01118065	[117]
	Everolimus (mTOR inhibitor)	Advanced follicular-derived TC (mainly DTC)	35 patients (28 DTC)	None (single arm)	Disease Control Rate (CR + PR + SD>24w)	DCR 58% (SD>24w); median PFS 9 mo; median OS 18 mo	Phase II Clinical Trial	NCT01118065	[116]
Immunotherapy & Combinations	Pembrolizumab + KI Salvage	ATC (post-KI progression)	12 patients	None (retrospective)	Best Overall Response, PFS, OS	BOR: 5 PR, 4 SD; Clinical Benefit Rate 75%	Low (Retrospective)	NCT03181100	[154]
	Lenvatinib + Pembrolizumab	Metastatic ATC and PDTC	8 patients	None (retrospective)	Best Overall Response Rate	ATC: CR 66%; PDTC: PR 100%; median PFS 17.75 mo	Low (Retrospective, small sample)	-	[134]

### The signaling-epigenetic-metabolic axis: a self-reinforcing oncogenic circuit

The limitations of single-target therapy and the theoretical basis of combination treatment strategies all originate from a precise, dynamic, and self-reinforcing triangular regulatory network in tumor cells - this network covers DNA methylation, metabolic reprogramming, and oncogenic signaling pathways. A deeper understanding of the causal interactions within this network is critical for the design of effective synergistic therapies.

Oncogenic signaling pathways (such as MAPK, PI3K/AKT) are key drivers. For example, the BRAF V600E mutation continuously activates the MAPK/ERK signaling pathway, thereby upregulating DNMT1 expression. This will lead to hypermethylation and silencing of differentiation gene promoters (such as TSHR, NIS), thereby forming a “signal → epigenetic” axis and initiating radioiodine resistance and dedifferentiation processes [34, 35, 96]. At the same time, these activated pathways upregulate key metabolic enzymes through transcriptional regulation, thus “initiating metabolic reprogramming” [119–121]. The resulting metabolic remodeling establishes the “metabolism→epigenetic” regulatory axis. Metabolites function as epigenetic messengers: glutamine-derived α-KG is a TET cofactor controlling demethylation [78, 79]; serine-glycine one-carbon metabolism supplies the methyl donor S-adenosylmethionine (SAM), influencing DNMT activity [94, 122–124]; and the oncometabolite D-2-HG inhibits TET, promoting genome-wide hypermethylation [125–129]. Thus, metabolic reprogramming directly regulates the methylation “writers” and “erasers.” Conversely, DNA methylation acts as a stable epigenetic “memory” that “consolidates and amplifies” aberrant metabolic states, forming an “epigenetic → metabolism” feedback axis. It silences tumor suppressors (e.g., PTEN, RASSF1A), releasing inhibition on signaling pathways and indirectly sustaining metabolic reprogramming [28, 130, 131]. It also directly targets metabolic genes - activating fatty acid synthase (FASN) through hypomethylation to promote lipid synthesis [88] or silencing fatty acid synthase 111B (FAM111B) through hypermethylation to enhance glycolysis [30] - thereby ensuring the long-term stability of tumor-promoting metabolism. These interactions form a self-perpetuating oncogenic circuit: “signaling activation → epigenetic remodeling → metabolic reprogramming → signal reactivation.” For instance, BRAF mutation drives MAPK activation, upregulates DNMT1, and silences TSHR/NIS. Resulting metabolic changes (e.g., increased SAM) further alter methyltransferase activity, silencing additional tumor suppressors, which in turn amplifies metabolic dysregulation and oncogenic signaling. This positive feedback loop is the main driving factor for the malignant development of tumor cells and the primary reason for the rapid development of drug resistance in single-target therapies [94, 131]. Therefore, future precision therapies must go beyond strategies targeting individual nodes. Their design should be based on an in-depth understanding of the causal logic of this network, and target two or more components within the “signal - epigenetic - metabolic” axis. Only through this collaborative strategy can this self-reinforcing cycle be broken, the malignant phenotype reversed, and treatment resistance overcome.

### Synergistic integration and challenges in immunotherapy

In TC, the immunosuppressive tumor microenvironment is jointly formed by the “DNA methylation-metabolism axis”, which provides a molecular theoretical basis for combining immunotherapy with epigenetic and metabolic interventions. Therefore, the research direction has shifted from the sole PD-1/PD-L1 treatment to a combined treatment strategy with targeted drugs. By disrupting the methylation and metabolic pathways, these targeted drugs not only directly induce tumor cell death but also indirectly alleviate the immunosuppression driven by epigenetics and metabolism, thereby reshaping the microenvironment to support anti-tumor immunity [132, 133].

The therapy is showing significant promise in clinical studies. For example, lenvatinib combined with pembrolizumab achieved an objective response rate of 66.7% in the treatment of undifferentiated and poorly differentiated thymic cancer, extending the median overall survival from 3–5 months in historical data to 17.3 months [134]. However, immune-related adverse events (such as hypertension, fatigue, and loss of appetite) and acquired drug resistance remain major challenges [134]. Future strategies could enable deeper integration through biomimetic nanotechnology, which enables precise and simultaneous delivery of epigenetic, metabolic, and immunomodulatory drugs. Combined with dynamic monitoring of circulating tumor DNA methylation to identify patients most likely to benefit, this integrated approach has the potential to overcome drug resistance and further improve efficacy.

### Innovative delivery system

Targeted drug delivery systems based on nanotechnology provide a key strategy to solve the pharmacokinetic bottleneck and systemic toxicity issues in multi-pathway combination therapy of radioiodine-refractory differentiated thyroid cancer (RAIR-DTC) [135]. By enhancing tumor-specific drug delivery, these systems aim to reduce off-target effects and improve the epigenetic drug therapeutic index. For instance, the clinical application of decitabine is often limited by toxicities such as bone marrow suppression in its conventional form. Encapsulating the drug in functionalized nanocarriers (such as liposomes or recombinant apolipoprotein B lipoproteins, rABLs) enables extensive tumor penetration and controlled release, thereby significantly reducing non-targeted organ toxicity, which has been confirmed in gastric cancer models [136].

More complex combined drug delivery systems further expand the treatment possibilities. For example, hexamethonium metal assemblies (HmA) combined with decitabine and nimigrelisin can induce tumor cell pyroptosis and activate systemic anti-tumor immunity [137]. Similarly, by using nanocarriers in combination with decitabine and MEK inhibitor temsirolimus, the synthetic lethal effect can be enhanced in the uveal melanoma model [138]. Moreover, the interaction between epigenetic regulation and immune activation is being exploited; a pH-responsive nanoplatform simultaneously delivers immunomodulatory siRNAs and histone modification inhibitors, successfully activating the cGAS-STING pathway, reprogramming T cells, and maturing dendritic cells, and exerting an effect in mice models resistant to PD-L1 [139]. These examples indicate that intelligent nanoplatforms can simultaneously deliver traditional epigenetic regulators (such as DNA methylation inhibitors) and immunotherapeutic agents to synergistically reshape the tumor immune microenvironment.

Although the above experimental data mainly come from non-TC cell models, they lay the foundation for treatment modalities applicable to tumor cells. Future efforts to develop specific biomimetic nanosystems targeting tumor cells for precise combined delivery of immunomodulators, signaling pathway inhibitors, and epigenetic drugs have great potential. Such a comprehensive strategy can simultaneously achieve the three key goals of tumor microenvironment remodeling, signaling pathway inhibition, and epigenetic reprogramming, potentially paving the way for efficient and low-toxicity combined therapies for advanced thyroid cancer.

### Clinical pathway for redifferentiation therapy based on DNA methylation

**Fig. 5:**
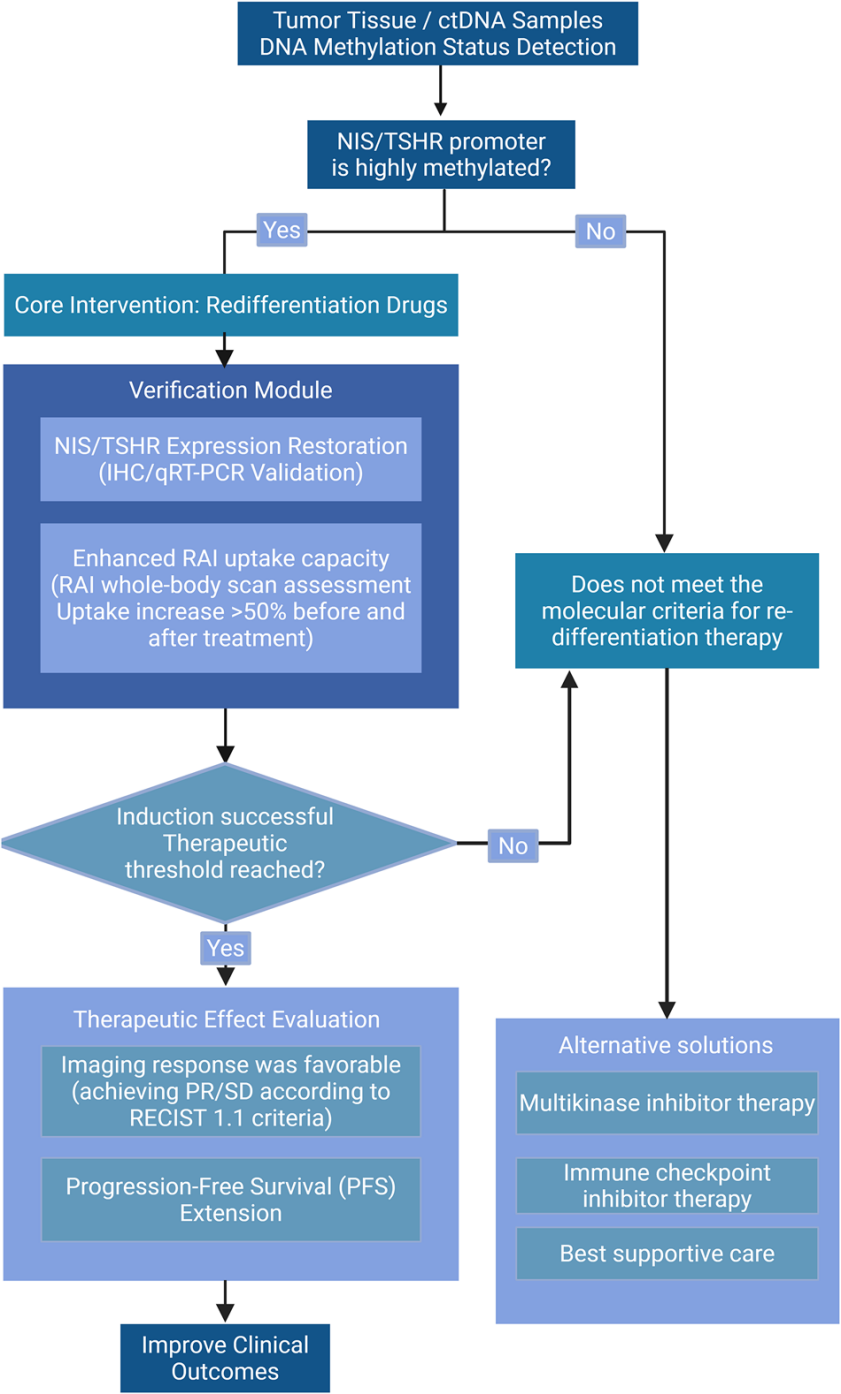
Concept diagram of clinical pathways for differentiated treatment of thyroid cancer based on NIS/TSHR promoter methylation status. This figure illustrates the treatment decision-making process using promoter methylation status as a molecular biomarker. This process is initiated when significant hypermethylation of the NIS/TSHR promoter region is detected in tumor tissue or circulating tumor DNA. A positive test triggers a redifferentiation regimen based on epigenetic drugs (e.g., DNMT or HDAC inhibitors) and/or signaling pathway inhibitors (e.g., BRAF or MEK inhibitors). Verification of treatment efficacy is based on two key indicators: restoration of NIS/TSHR protein expression and significant improvement in radioactive iodine (RAI) uptake. The final efficacy evaluation follows the RECIST 1.1 standard and is determined by objective response rate and progression-free survival (PFS). For patients who are negative for methylation markers or who fail to recover function after treatment, alternative treatment options should be considered. NOTE: Quantitative thresholds for “significant” hypermethylation and improvement in radioactive iodine uptake may vary depending on assay method and study design; the core principle is to confirm clinically meaningful functional reversal. Data Source: [35, 36, 38, 39, 94, 134, 142–146, 148]. Created with BioRender.com.

Based on the “DNA methylation-metabolism axis” theory and the established role of DNA methylation in the dedifferentiation and treatment resistance of thyroid cancer, this study proposes a clinical pathway for re-differentiation therapy, aiming to provide a decision-making framework for the precise treatment of RAIR-DTC (Fig. 5).

This pathway begins with the accurate determination of the methylation status of key gene promoters (such as NIS and TSHR) in tumor tissues or circulating tumor DNA (ctDNA) using high-sensitivity techniques such as pyrosequencing or methylation-specific PCR [140, 141]. The results show that patients with significantly high promoter methylation - indicating that iodine uptake disorder is likely driven by epigenetic silencing - constitute the main target population for re-differentiation therapy.

To reverse abnormal epigenetic silencing, the core intervention strategies include the direct use of epigenetic regulators, such as DNA methyltransferase inhibitors (e.g., decitabine) or histone deacetylase inhibitors (e.g., sodium butyrate). Additionally, the oncogenic signaling pathways (such as BRAF or MEK inhibitors dabrafenib/ selumetinib) can be inhibited to indirectly restore thyroid-specific gene expression. These methods can be used alone or in combination [36, 94, 142–144]. The treatment response needs to be verified by two objective criteria: (1) Immunohistochemistry or qRT-PCR confirms the restoration of NIS/TSHR protein and mRNA expression; (2) The comparison of whole-body radioiodine (RAI) scans before and after treatment shows a significant improvement in RAI uptake function [36, 144]. Iodine-131 has a dual role in this treatment pathway. Conventional radioactive iodine therapy is ineffective in the initial RAIR state, but successful redifferentiation can restore iodine-131 affinity, making the patient eligible for radical or palliative radioactive iodine therapy again. Therefore, the primary goal of this pathway is to restore iodine-131 uptake function. After functional recovery, patients entered the standard radioactive iodine treatment and follow-up phase. The final efficacy was evaluated based on the RECIST 1.1 standard [145, 146] and confirmed clinical endpoints such as objective response rate and progression-free survival. Patients who do not develop significant promoter hypermethylation or do not regain function after redifferentiation therapy may have complex resistance mechanisms involving other pathways. Alternative treatment options—including immune checkpoint inhibitors, multikinase inhibitors, and optimized supportive care—should be considered for such patients [134, 147, 148]. In summary, this clinical pathway transforms basic research results into a systematic framework for RAIR-DTC management, follows the logic of “molecular diagnosis guides intervention and functional imaging verifies efficacy”, and provides research ideas for the implementation of personalized precision treatment.

## Conclusion and future prospects

The concept of “DNA methylation-metabolism axis” proposed in this review reveals a previously unidentified self-reinforcing pathogenic cycle at the core of thyroid cancer (TC). This comprehensive framework not only integrates evidence of metabolic reprogramming and epigenetic dysregulation, but also fundamentally redefines TC progression and treatment resistance as a feedback imbalance disease. Thus, it shifts the paradigm of rational treatment design from a sequential or single-target approach to simultaneously targeting metabolic and epigenetic nodes in this cycle. This new perspective highlights key research gaps and directions for future exploration.

Several key scientific questions arise in the current study and deserve further exploration. First, whether metabolites (such as lactic acid, α-ketoglutarate) regulate TET/DNMT activity through a cooperative mechanism and achieve spatiotemporal specificity remains to be further elucidated [43, 149]. Secondly, it is necessary to determine whether key metabolic enzymes, such as stearoyl-CoA desaturase 1 (SCD1) and phosphoglycerate dehydrogenase (PHGDH), may become potential network hubs because these enzymes can form a positive feedback loop to enhance radioactive iodine (RAI) tolerance [47, 54, 85]. Third, it is necessary to further analyze the precise regulatory network that suppresses signaling pathways (such as BRAF/MEK/ERK pathways) and enhances glycolysis gene expression through DNA methylation (inhibiting genes such as TSHR), thereby promoting a self-reinforcing cycle mechanism. This area remains to be further elucidated [34, 35, 120].

Translating this axis into therapeutic strategies holds great promise but also faces significant challenges. It is a promising strategy to develop biomimetic nanocomposite delivery systems (such as liposomes, pH-responsive carriers) to simultaneously deliver drugs such as decitabine and MEK inhibitors. This system aims to enhance target specificity, while intervening in immune microenvironment remodeling, signaling pathway inhibition, and epigenetic reprogramming, which may reverse T cell exhaustion, activate the cGAS-STING pathway, and synergistically induce pyroptosis in tumor cells [137–139].

However, this framework faces numerous significant obstacles in clinical application. From a mechanistic perspective, the spatiotemporal specificity of epigenetic regulation mediated by metabolites and its variations in different tumor cell subtypes remain unclear. From a practical perspective, the lack of predictive biomarkers for evaluating the efficacy of combined treatments, methods to overcome resistance, and approaches to reduce off-target toxicity are major obstacles. From a technical perspective, there is an urgent need for advanced disease models that are closer to the human tumor microenvironment and tools that can simultaneously track the dynamic interactions between DNA methylation and metabolic reprogramming.

Future research should focus on three priority areas: First, single-cell multi-omics platforms can be used to deeply study the dynamic regulatory patterns within the DNA methylation-metabolism network. Second, developing innovative bionic drug delivery systems for the precise co-delivery and controlled release of immunomodulatory, metabolic, and epigenetic agents. Third, establishing sophisticated preclinical models, such as patient-derived organoids and humanized tumor xenografts, to accelerate the clinical translation of multimodal combination therapies targeting this axis. Through these concerted efforts, we aim to solidify this novel theoretical framework and translate it into effective therapeutic strategies for treatment-resistant TC.

## Data Availability

Not Applicable.
